# The effects of ventilation tubes versus no ventilation tubes for recurrent acute otitis media or chronic otitis media with effusion in 9 to 36 month old Greenlandic children, the SIUTIT trial: study protocol for a randomized controlled trial

**DOI:** 10.1186/s13063-016-1770-x

**Published:** 2017-01-19

**Authors:** Malene Nøhr Demant, Ramon Gordon Jensen, Janus Christian Jakobsen, Christian Gluud, Preben Homøe

**Affiliations:** 1 Department of Otorhinolaryngology and Maxillofacial Surgery, Zealand University Hospital, Køge, Denmark; 2 Copenhagen Trial Unit, Centre for Clinical Intervention Research, Rigshospitalet, Copenhagen University Hospital, Copenhagen, Denmark; 3 Department of Cardiology, Holbæk Hospital, Holbæk, Denmark; 4 Institute of Clinical Medicine, Faculty of Health and Medical Sciences, University of Copenhagen, Copenhagen, Denmark

**Keywords:** Otitis media, Randomized clinical trials, Ventilation tubes, Grommets

## Abstract

**Background:**

The prevalence of otitis media in Greenlandic children is one of the highest in the world. International studies have shown that otitis-prone children may benefit from tubulation of the tympanic membrane. However, it is unknown whether these results can be applied to Greenlandic children and trials on the effects of ventilation tubes in high-risk populations have, to our knowledge, never been conducted.

**Methods:**

The trial is an investigator-initiated, multicentre, randomized, blinded superiority trial of bilateral ventilation tube insertion versus treatment as usual (no tube) in Greenlandic children aged 9–36 months with chronic otitis media with effusion or recurrent acute otitis media. With randomization stratified by otitis media subtype and trial site, a type 1 error of 5% and a power of 80%, a total of 230 participants are needed to detect a decrease of two visits to a health clinic during 2 years, which is considered the minimal clinical relevant difference. The primary outcome measure will be assessed blindly by investigating medical records. Secondary outcome measures are number of episodes of acute otitis media, quality of life, number of episodes of antibiotics administration and proportion of children with tympanic membrane perforations.

**Discussion:**

This trial will provide evidence-based knowledge of the effects of ventilation tubes in children with middle ear infections from the high-risk Greenlandic population. Furthermore, this trial will improve the understanding of conducting randomized clinical trials in remote areas, where management of logistical aspects is particularly challenging.

**Trial registration:**

ClinicalTrials.gov, NCT02490332. Registered on 14 February 2016.

**Electronic supplementary material:**

The online version of this article (doi:10.1186/s13063-016-1770-x) contains supplementary material, which is available to authorized users.

## Background

Otitis media is one of the most common reasons for children to contact health clinics and insertion of ventilation tubes in the tympanic membrane remains the most frequent type of paediatric surgery in the USA as well as the main reason for prescription of out-of-hospital antibiotics to paediatric patients [1–3]. The worldwide socioeconomic consequences of the disease are substantial, owing to treatment and management of the disease and parental absence from work [4].

The prevalence of otitis media and other acute respiratory tract infections in Greenlandic children is one of the highest in the world; 20% of schoolchildren have impaired hearing in the frequencies of normal speech [5–10]. This pattern is also seen in, for example, the indigenous population in Australia [11]. Many theories have been suggested to explain the high prevalence in certain indigenous populations, such as anatomical features, poverty or limited access to health-care, as well as a high bacterial load in the nasopharynx [12–15]. Studies have shown that the prevalence of chronic suppurative otitis media in the two largest towns in Greenland, Nuuk and Sisimiut, is 9% to 14%, which is a public health problem requiring urgent attention, according to the World Health Organization [6, 7, 12]. A total of 91% of children with chronic suppurative otitis media develop permanent hearing loss, which underlines the importance of treatment and management of the disease [9].

Previous studies have shown that risk factors for the development of chronic suppurative otitis media are associated with the number of upper respiratory tract infections, as well as attendance of day care, mothers’ educational status, passive smoking and socioeconomic factors, similar to known risk factors for acute otitis media found in other studies worldwide [7, 8, 10, 16]. It has been suggested that children with chronic otitis media with effusion, as well as recurrent acute otitis media, are more prone to develop chronic suppurative otitis media, and treatment of these conditions might therefore decrease the number of children with chronic suppurative otitis media [6, 17]. The cumulative incidence of chronic suppurative otitis media in Greenland is 14% at the age of 4, with the highest hazard rate between 6 and 12 months [7]. This indicates that the disease develops early in childhood, and calls for intervention as early as possible in otitis-prone children, to limit progression to chronic perforations.

Ventilation tubes are inserted in the tympanic membrane to equalize pressure and allow drainage of middle ear fluid.

According to guidelines from the UK, the USA and Denmark [1, 3, 18–21], there are two indications for the insertion of ventilation tubes in children:

- Chronic otitis media with effusion and hearing loss
- Recurrent acute otitis media

Chronic otitis media with effusion is defined as fluid in the middle ear cavity lasting ≥3 months. Recurrent acute otitis media is defined as ≥3 episodes of acute otitis media within 6 months or ≥4 episodes of acute otitis media within 12 months [21].

Guidelines on the treatment of otitis media with effusion are fairly similar and have been recently updated in both the USA and the UK [3, 20]. However, guidelines on treatment of recurrent acute otitis media vary and different guidelines currently exist in the USA, while there are no national guidelines in the UK [1, 19, 21]. This might explain part of the observed international differences in the number of ventilation tube insertions for recurrent acute otitis media [22].

The mentioned international guidelines on treatment with ventilation tubes are generally based on evidence of low methodological quality concerning such outcomes as number of otitis media episodes after treatment, quality of life after treatment, reduction in chronic tympanic membrane perforations after treatment and number of episodes of aural discharge after treatment, according to the GRADE evaluation of quality of evidence [18, 21, 23, 24]. Furthermore, it is primarily children from Western trial populations, and not children from high-risk otitis-prone populations, such as the Inuit in Greenland, who have been randomized in the previous trials. Currently, there are no national guidelines or programmes to ensure prevention and treatment of otitis media and impaired hearing in Greenland. The Greenlandic Ministry of Health has considered introducing ventilation tube insertion as a more consistent treatment modality in order to decrease the burden of otitis media in the country. However, trials on the effects of ventilation tubes among children in high-risk populations have, to our knowledge, never been conducted. We therefore argue that it is both medically and ethically necessary to conduct a randomized clinical trial before ventilation tubes are made part of the routine treatment of children with chronic otitis media with effusion and recurrent acute otitis media in Greenland. Moreover, ventilation tube treatment is currently not a part of standard care in Greenland and this provides a unique opportunity to investigate the unbiased effect of the treatment, which would not be possible in populations where ventilation tube treatment is already standard treatment.

## Methods/Design

### Objective and hypothesis

The primary objective of the trial will be to assess the effects of bilateral insertion of ventilation tubes versus ‘treatment as usual’ with no ventilation tubes in children 9–36 months old with chronic otitis media with effusion or recurrent acute otitis media in Greenland, measured by number of visits to health clinic for 2 years after randomization, assessed by investigating medical records.

The null hypothesis is:

- There is no difference in the number of visits to health clinics for children with chronic otitis media with effusion or recurrent otitis media treated with bilateral ventilation tubes, compared with children not treated with ventilation tubes.

### Design

We have designed an investigator-initiated, parallel-group, multicentre, randomized clinical superiority trial of bilateral ventilation tube insertion versus treatment as usual (no ventilation tube) in Greenlandic children with chronic otitis media with effusion or with recurrent acute otitis media.

**Fig. 1:**
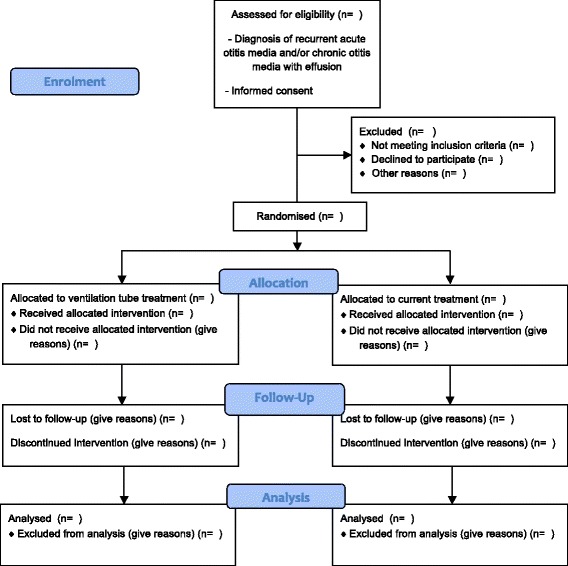
Consolidated Standards of Reporting Trials (CONSORT) trial flow chart

The Consolidated Standards of Reporting Trials (CONSORT) flow chart for the trial is shown in Fig. 1 [25]. The Standard Protocol Items: Recommendations for Interventional Trials (SPIRIT) participant timeline is given in Table 1 and the SPIRIT checklist is given in Additional file 1 [26].

**Table 1:** Participant timeline, Standard Protocol Items: Recommendations for Interventional Trials (SPIRIT) diagram

	Study period					
Time point	Enrolment	Allocation				Close-out
Enrolment:	−*t*_1_	0	*t*_1_	*t*_1_ + 3 months	*t*_1_ + 1 year	*t*_1_ + 2 years
Eligibility screen	X					
Informed consent	X					
Allocation	X					
Interventions:		X				
Ventilation tube insertion			X		X	
No ventilation tube insertion			X		X	
Assessments:						
Sex, age, ethnicity, socioeconomic factors	X					
Number of visits to health clinic						X
Questionnaires: Otitis Media-6 and Caregiver impact		X	X	X	X	X
Safety variables						X

We will consider all patients followed at a participating trial site for participation and include patients if they comply with the inclusion and exclusion criteria listed in Table 2.

**Table 2:** Inclusion and exclusion criteria

Inclusion criteria	Exclusion criteria
Children aged 9–36 months	Children with orofacial cleft
Children with at least one Greenlandic born parent and at least one Greenlandic born grandparent	Children with Down’s syndrome
American Society of Anaesthesiologists’ physical status classification class 1 and 2	Children with known generalized immune deficiency
B-type curve, defined as flat line tympanograms or gradient <0.04 ml, or C2-type curve, defined as pressure ≤ −200 dPa, measured by tympanometry at two visits three-four months apart or	Children formerly treated with ventilation tubes
three episodes of acute otitis media in 6 months according to medical charts or	Lack of signed informed consent, signed by the legal guardian
tour episodes of acute otitis media in 12 months according to medical charts	
Signed informed consent, signed by the legal guardian	

**Fig. 2:**
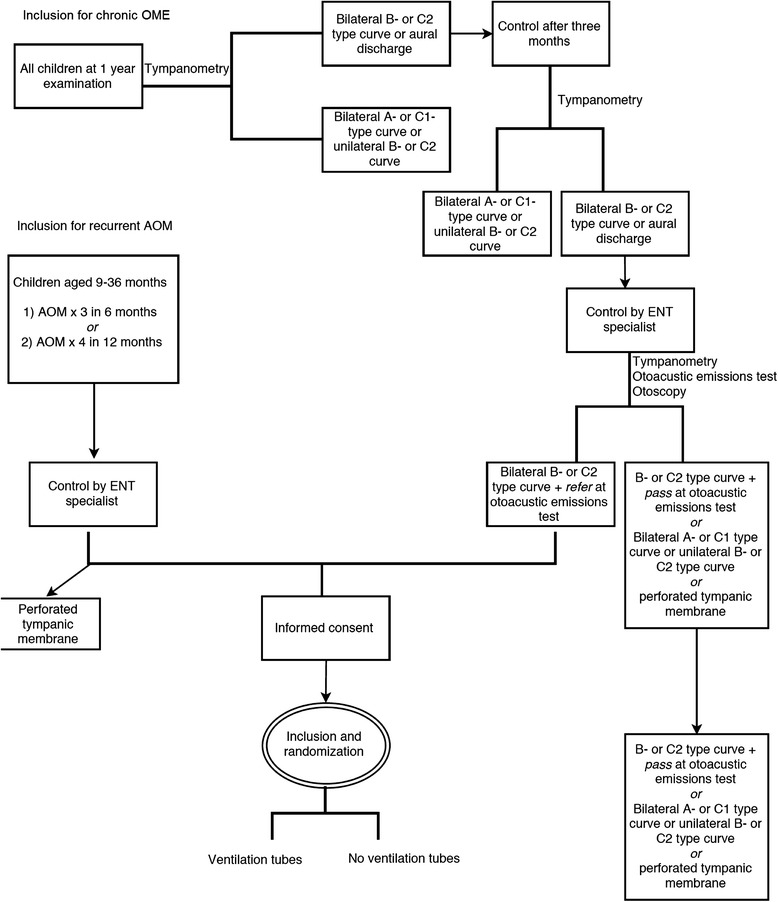
Inclusion procedure flowchart. AOM, acute otitis media; ENT, ear, nose and throat; OME, otitis media with effusion

The inclusion procedure is shown in Fig. 2.

We will include children aged 9–36 months. Infants younger than this age group require special anaesthesiological care, which cannot be offered in Greenland for ventilation tube insertion. The upper age limit has been set as low as possible in order to intervene before progression of the disease, while ensuring adequate sample size in this small population. We have attempted to reduce the number of exclusion criteria to as few as possible. However, children with orofacial cleft, Down’s syndrome and immune deficiency are, for other reasons, known to be at very high risk of chronic otitis media with effusion and recurrent acute otitis media and are therefore not comparable with other children. Children already treated with ventilation tubes can also not be included.

### Trial site and personnel

The trial sites are hospitals and health clinics in six Greenlandic towns: Nuuk, Sisimiut, Ilulissat, Aasiaat, Qaqortoq and Tasiilaq. The Greenlandic health-care system is divided into primary and secondary sectors. The only large and secondary referral hospital is in the capital, Nuuk [27].

The personnel performing the initial selection and screening of participants will be the regular staff at the Greenlandic health clinics and hospitals, for instance nurses, assistants and doctors of different specialities and ranks.

### Randomization

The enrolment of patients will be conducted by an ear, nose and throat (ENT) specialist and the coordinating investigator. We will use centralized stratified web-based randomization. Prior to randomization, a computer will generate randomization sequences with varying block sizes that are unknown to the investigators. An internet-based randomization system will be set up conducting randomization stratified according to centre (trial site) and type of otitis media, i.e., recurrent acute otitis media or chronic otitis media with effusion at baseline (yes or no). The randomizing investigator will access the internet site through a personal information number. Patients who meet both criteria for chronic otitis media with effusion and recurrent acute otitis media will be considered in the recurrent acute otitis media group. The patients will be randomly allocated 1:1 into the two intervention groups.

### Interventions

The experimental intervention consists of bilateral insertion of ventilation tubes (Donaldson) in the tympanic membranes, administered under general anaesthesia.

Short-term ventilation tubes will be used, consistent with the tube type applied in the majority of other studies, and in accordance with the type that is expected to be introduced in Greenland [18, 23]. If the tympanic membrane is infected at the time of ventilation tube insertion, topical antibiotics will be given (Cilodex® [dexamethasone + ciprofloxacin] eardrop suspension, 3 mg + 1 mg, dexamethasone + ciprofloxacin) at a dose of four drops twice daily for 5 days.

If children in the intervention group seek medical assistance for ear problems after the insertion of ventilation tubes, these ear problems will be treated according to current practice in Greenland, which includes systemic antibiotic treatment (amoxicillin 40–90 mg/(kg day)), as well as aural toilette and topical antibiotics (ciprofloxacin 1 ml/3 mg, three drops, twice daily, or Cilodex® [dexamethasone + ciprofloxacin] eardrop suspension, 3 mg + 1 mg, four drops twice daily for 5–7 days).

The control intervention will be based on the current practice in Greenland, which includes systemic antibiotic treatment (amoxicillin, 40–90 mg(kg day)), as well as aural toilette and topical antibiotics (ciprofloxacin 1 ml/3 mg, three drops, twice daily). Children in the control group will not be offered ventilation tubes for any circumstance until at least 2 years after the first ENT visit and randomization.

Children in both the intervention group and control group will have an ENT examination, including otoscopy by an ENT specialist at least once a year and at the end of the study period, 2 years after randomization.

### Outcomes

To assess the primary outcome in as unbiased a manner as possible, it is necessary to choose an outcome that does not require visualization of the tympanic membrane, as this might reveal the trial intervention allocation of the patient (the ventilation tube or sequelae to such a tube would be visible). Therefore, the primary outcome will be the number of visits to health clinics during 2 years after randomization, determined according to the medical records, assessed blinded to intervention. In otherwise healthy children, the number of visits to health clinics can be assumed to reflect the number of episodes related to the ear or upper respiratory tract, as this is thought to be the primary reason for contact to health clinics for children aged 9–36 months [28].

It is currently not possible to assess hearing level as an outcome measure because there are no facilities available in Greenland able to meet the high standard of hearing evaluation, which would be necessary to detect differences in hearing levels of 4 dB [23]. We therefore postulate that an effect of ventilation tubes on otitis media with effusion might be reflected in a change in the number of visits to health clinics. The number of episodes of acute otitis media, based on medical records, will be included as a secondary outcome measure but will not be used as a primary outcome measure because the treatment providers and the outcome assessors will often not be sufficiently blinded to the trial intervention allocation.

#### Primary outcome measure

This is the number of visits to health clinic during 2 years after the randomization, based on medical records, evaluated by designated assessors blinded to the intervention.

#### Secondary outcome measures

1. Number of episodes of acute otitis media during the 2 years after the randomization, based on medical records, evaluated by designated assessors blinded to the intervention
2. Quality of life, measured on a 0–100 scale by the validated Otitis Media-6 questionnaire [29, 30] and the Caregiver Impact Questionnaire [31, 32], assessed at randomization, 3 months after randomization, 1 year after randomization and at the end of the trial, 2 years after randomization
3. Number of episodes during the 2 years after randomization where oral or intravenous antibiotics have been administered, based on medical records, evaluated by designated assessors blinded to the intervention
4. Proportion of children with unilateral or bilateral tympanic membrane perforations in the intervention and control group at the end of the trial 2 years after randomization, based on otoscopical photos, which will be anonymized and evaluated by an ENT specialist without knowledge of the intervention
5. Serious adverse events during the 2 years after the randomization: any adverse event that results in death, is life threatening, requires hospitalization or prolongation of existing hospitalization or results in persistent or significant disability or incapacity [33]

#### Exploratory outcomes

1. Number of episodes of aural discharge during the 2 years after randomization, based on medical records, evaluated by designated assessors blinded to the intervention

The primary outcome measure as well as secondary outcome measures 1 and 3 and the exploratory outcome measure will be based on medical records, while secondary outcome measure 4 will be based on clinical examination. Secondary outcome measure 2 will be based on questionnaires; the first questionnaires will be completed at the clinical examination related to randomization, the remaining questionnaires will be sent to the participants’ legal guardians by email and completed online.

### Blinding

Owing to the type of the intervention, blinding of patients, parents and caregivers is not possible. However, the outcome assessors will be blinded to intervention and we also consider the number of visits to health clinics according to medical records (the primary outcome measure) as blinded. Outcome assessors will be ENT specialists.

Blinding of the number of visits at the health-care centre is ensured by initial blinding of medical records by an investigator, and hereafter evaluation by two outcome assessors calculating the number of visits from the medical records. A third assessor may provide further evaluation in the event of any disagreement. Blinding of otoscopical results is ensured by the use of otoscopical photos, which will be anonymized and evaluated by an ENT specialist without knowledge of the intervention.

Blinding of quality-of-life measures cannot be obtained as the child and the parents are not blinded for the intervention. Therefore, this outcome measure must be considered at high risk of bias.

### Assessment of adverse events

Adverse events and adverse reactions will be assessed at every ENT visit.

### Participant discontinuation and withdrawal

Parents of participating children can withdraw their consent to participate at any time. To be able to analyze data at an intention-to-treat basis the investigator must ask for permission to use already collected data for data analysis.

The investigator or treating physician may discontinue the patient from further participation in the trial if the patient is diagnosed with any of the exclusion criteria. The investigator and treating physician will encourage the patient to continue the follow-up assessment and previously collected data should be used in further analysis.

We will monitor adherence to the control regimen. Parents of participating children will be reminded, at the time point for intervention and assessments, by email. Those who do not adhere to the control regimen specified will be further contacted by phone call and email.

### Data management

Data will be entered in the data management system Easy Trial. Easy Trial hold standards according to the Danish Data Protection Agency, i.e., data are stored on private servers. Case report forms in electronic format will be used, as well as case report forms on paper. The paper case report forms will be entered in the data management system twice by two personnel independently to promote data quality. The personnel will otherwise not be related to the trial. Patients will be identified by patient identification number, which is also used at randomization. The trial is conducted according to regulations by the Danish Data Protection Agency and only people related to the trial and the central randomization centre will have access to data.

Missing data will be minimized by checking the completed questionnaires when returned to the investigators. If there are any missing answers, the parents of the included children will emailed a request to supply the missing answers.

### Statistical plan and data analysis

Based on power (1 − *β*) = 0.80, *α* = 0.05 (two-sided) and standard deviation of five visits to health clinics and

- An estimated eight visits to health clinics during 2 years in the control group and
- An estimated six visits to health clinics during 2 years in the experimental group

we need a sample size of 99 individuals in each intervention group.

As we do not expect that data are normally distributed, the non-parametric van Elteren test will be used; thus, we obtain 99/0.86 = 115 participants per intervention group or 230 participants in total [34].

The two interventions will be compared regarding all outcomes. The analysis of the outcomes will be based on the intention-to-treat principle, i.e., all randomized participants will be included in the analysis, regardless of how much treatment they have received. Per-protocol analysis may be considered if important deviations from the protocol compromise the validity of the intention-to-treat analysis.

Dichotomous outcomes will be analyzed using logistic regression, continuous outcomes will be analyzed using linear regression and count data will be analyzed using the van Elteren test [34]. Our primary analysis will be adjusted for the stratification variables used in the randomization (trial site and type of otitis media). In secondary analysis, we will adjust all analyses (except when non-parametric tests are used) for additional significant design variables (age, sex, attending daycare, smokers in the household, diet, family history of otitis media). The statistical analysis will be described in detail in a separate paper published before the analysis of the trial results begins.

If only data are missing on the dependent outcome, we will use per-protocol data but we will interpret out results with caution if these missing data potentially bias our results. Otherwise, if more than 5% of the outcome data are missing, multiple imputation will be used (STATA 14). However, the 5% cut-off is not definitive. The imputation result will be considered the primary overall result. This analysis will be supplemented by the following sensitivity analyses:

1. **‘Best-worst-case’ scenario:** It will be assumed that all participants lost to follow-up in the experimental group have a mean score +2 standard deviations and have no event; and all those with missing outcomes in the control group have a mean score of −2 standard deviations and have an event.
2. **‘Worst-best-case’ scenario:** It will be assumed that all participants lost to follow-up in the experimental group have a mean score of −2 standard deviations and have an event; and all those lost to follow-up in the control group have a mean score +2 standard deviations and have no event [35].

Results from both scenarios will be presented in our trial publication.

If the null hypotheses on the primary outcome measures are not rejected, our main conclusion will be that we found no significant difference between the two interventions. The analysis of the remaining outcome measures will be presented for hypothesis-generating purposes.

## Discussion

This trial will provide evidence-based knowledge of the effects of ventilation tubes in children with middle ear infections. Furthermore, the effects of ventilation tube administration in a high-risk population, such as the Greenlandic, have never, to our knowledge, been investigated and this trial will improve the understanding of conducting randomized clinical trials in remote areas, where management of logistical aspects is particularly challenging.

The strengths of this trial are the inclusion of children at high risk of developing otitis media and sequelae thereof; the central randomization regarding both generation of allocation sequence and allocation concealment; the primary outcome measure that monitors use of the health-care system; and our attempts to blind as many outcome measures as possible. Our trial also has limitations. First, no updated systematic review of the effects of ventilation tubes is currently available. We refer to previous Cochrane reviews published in 2008 and 2010, respectively [18, 23] but the methodology of these reviews is not optimal and the literature search has not been sufficiently updated. We are writing a protocol for a systematic review assessing the effects of ventilation tubes; this protocol will be registered on PROSPERO. As soon the protocol is registered, we will perform a literature search and begin writing the review. Nevertheless, it is a major methodological limitation that we cannot sufficiently take into account a complete and valid overview of previous studies on the effects of ventilation tubes. Further methodological limitations are a lack of blinding to the intervention regarding a number of the outcome measures and potential problems with drop-out during follow-up, owing to lack of interest, migration or logistics.

### Dissemination policy

The Greenlandic population will be informed of the trial as well as its final results through national media. All participating health clinics and hospitals will be visited by the coordinating investigator and instructed in objectives and screening or inclusion procedures. The final and interim results will be presented at NUNA MED, an international conference on Greenlandic medicine and health held every third year.

Trial results will be published in English, Danish and Greenlandic.

The Government of Greenland will be informed of the final results before a press release is issued but will have no influence on the reporting of the results.

## Trial status

We launched the randomization on 18 February 2016. At the end of June, six children had been enrolled and randomly allocated to a group.

## Additional file

Additional file 1: SPIRIT 2013 Checklist. (DOCX 61 kb)

## Abbreviation

CONSORT: Consolidated Standards of Reporting Trials
ENT: ear, nose and throat
SPIRIT: Standard Protocol Items: Recommendations for Interventional Trials
